# TWIST1 induces proteasomal degradation of β-catenin during the differentiation of ovarian cancer stem-like cells

**DOI:** 10.1038/s41598-022-18662-2

**Published:** 2022-09-19

**Authors:** Jiaqi Liu, Guang Shu, Anqi Wu, Xiaojun Zhang, Zhengwei Zhou, Ayesha B. Alvero, Gil Mor, Gang Yin

**Affiliations:** 1 Department of Pathology, School of Basic Medical Sciences, Xiangya Hospital, Central South University, Changsha, 410013 China; 2 C.S. Mott Center for Human Growth and Development, Department of Obstetrics and Gynecology, Wayne State University, Detroit, MI USA; 3 Department of Histology and Embryology, School of Basic Medical Sciences, Central South University, Changsha, 410013 China; 4 China-Africa Research Center of Infectious Diseases, School of Basic Medical Sciences, Central South University, Changsha, 410013 China; 5 National Clinical Research Center for Geriatric Disorders, Xiangya Hospital, Central South University, Changsha, 410008 China

**Keywords:** Oncogenes, Cancer microenvironment, Cancer, Cancer genetics

## Abstract

Ovarian cancer (OC) is one of the leading gynecologic cancers worldwide. Cancer stem-like cells are correlated with relapse and resistance to chemotherapy. Twist1, which is involved in ovarian cancer stem-like cell differentiation, is positively correlated with CTNNB1 in different differentiation stages of ovarian cancer cells: primary epithelial ovarian cancer cells (primary EOC cells), mesenchymal spheroid-forming cells (MSFCs) and secondary epithelial ovarian cancer cells (sEOC cells). However, the expression of β-catenin is inversed compared to CTNNB1 in these 3 cell states. We further demonstrated that β-catenin is regulated by the protein degradation system in MSFCs and secondary EOC but not in primary EOC cells. The differentiation process from primary EOC cells to MSFCs and sEOC cells might be due to the downregulation of β-catenin protein levels. Finally, we found that TWIST1 can enhance β-catenin degradation by upregulating Axin2.

## Introduction

Ovarian cancer is the most common cause of death from gynecologic cancers in the world. Every year, approximately 230 000 women are diagnosed worldwide, and 150 000 women die from the disease^1,2^. A main factor contributing to the high mortality rate is the lack of means for early diagnosis and early detection of recurrence^1,3^.

Cancer stem-like cells are unique cell population with the potential for self-renewal, metastasis formation and chemoresistance, thus supporting cancer progression^4–7^. There are strong evidences that ovarian cancer is driven and sustained by cancer stem-like cells^8,9^, these cells are root of cancer recurrence and therapy resistance^10–13^. Understanding the key features and mechanisms by which cancer stem-like cells maintain tumor growth and differentiation status provides an opportunity to improve patient prognosis^3^. Previous research has reported that mesenchymal cells that have undergone epithelial-mesenchymal transition (EMT) behave in many respects similarly to stem cells^14^. We have previously reported the identification of two populations of epithelial ovarian cancer (EOC) cells^15–26^, Type I and Type II. Type I/CD44^+^ EOC cells are associated with cancer stem cell (CSC) characteristics: (1) tumorigenic and can recapitulate the heterogeneity of the original tumor; (2) can form self-renewing spheroids; (3) have high levels of stem cell markers β-catenin, Oct-4, and SSEA-4; (4) have constitutively active IKKβ/NF-κB; (5) constitutively secrete IL-6, IL-8, MCP-1, and GROα; and (6) are chemoresistant^15^. We used these cells as “primary epithelial ovarian cancer cells (primary EOC cells)” in our study. Type II/CD44^-^ EOC cells are a differentiated population and are sensitive to chemotherapy^24^. “Mesenchymal spheroid-forming cells (MSFCs)” and “secondary epithelial ovarian cancer cells (sEOC cells)”, which were differentiated from “primary EOC cells”^25,26^.

The basic helix-loop-helix transcription factor TWIST1 is highly expressed in various types of human cancers^27,28^ and function as an oncogene^14,29,30^. We have reported that TWIST1 is an important regulator of “stemness” in epithelial ovarian cancer (EOC) cells. It is associated with the transition of epithelial stem-like Type I/CD44^+^ EOC cells to mesenchymal Type II/CD44^-^ EOC cells, suggesting that TWIST1 regulates ovarian cancer cell differentiation^25^. Many studies eported that TWIST1 could maintain the stemness of cancer cells and is necessary for cancer cell dissemination, proliferation and metastases ^31–34^. Wnt/β-catenin signaling is vital to all stages of tissue differentiation. The Wnt signaling pathway influences tumorigenesis in ovarian cancer^35,36^. The Wnt/β-catenin pathway regulates many critical facets of ovarian cancer development, including cancer cell proliferation, survival^37^, enhancing metastasis and tumor angiogenesis^38^, immune suppression^39^, and maintaining cancer stemness^40–47^. Under Wnt-off conditions, cytoplasmic β-catenin is degraded by the destruction complex composed of APC, Axin, GSK3β and CK1^48^. From 16 to 54% of endometrioid ovarian carcinomas and 14% of mucinous ovarian carcinomas have mutations in the CTNNB1 gene. Serous ovarian cancer and clear cell ovarian cancer have nuclear β-catenin accumulation^49–55^. After β-catenin accumulates and translocates to the nucleus, it binds the T cell factor/lymphoid enhancer-binding factor (TCF/LEF) family of transcription factors and drives the transcription of WNT target genes such as RNF43, ZNRF3, and LGR5^56–58^.

Since TWIST1 and β-catenin both play important role in cancer formation and progression, we hypothesized that TWIST1 may regulate β-catenin expression and function due to the correlation of TWIST1 and CTNNB1 in our EMT assay. In the present study, we demonstrated that the expression of TWIST1 is different in various differentiation processes from primary EOC cells to MSFCs and sEOC cells, meanwhile, TWIST1 enhances β-catenin degradation by upregulating Axin2.

## Results

### Expression of CTNNB1 and β-catenin and the potential mechanism in different ovarian cancer cell stages

**Figure 1:**
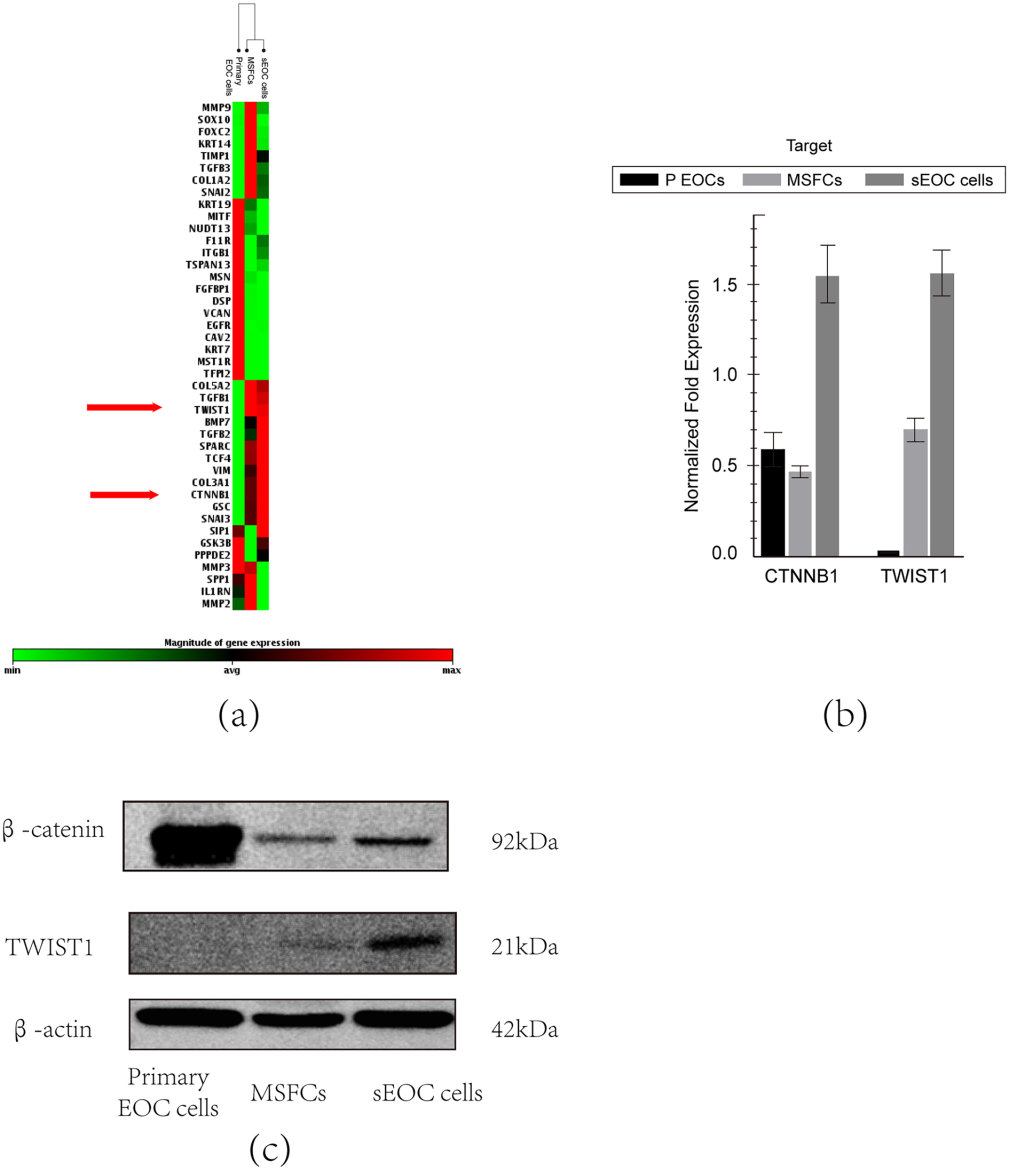
Expression of CTNNB1 and β-catenin in primary EOC cells, MSFCs and sEOC cells. (**a**)The EMT array showed that the transition from primary EOC cells (P EOC cells) to MSFCs and sEOC cells is characterized by the upregulation of genes associated with EMT and MET. Array was performed using 3 clones for each stage of differentiation; genes with *p* < 0.05 are shown. (**b**)qPCR analysis of CTNNB1 mRNA isolated from P EOCs, MSFCs and sEOC cells. Three replicates of each gene were performed for each experiment. Data are representative of three independent experiments. *p < 0.05, Student’s t test. (**c**)Western blot of β-catenin and TWIST1 protein expression in ovarian cancer cell lines. Data are representative of three independent experiments.

TWIST1 is degraded in epithelial ovarian cancer cells (EOC) to maintain their epithelial phenotype^20,25,26^. To explore how TWIST1 induces differentiation of EOCs, we have performed an EMT array with mesenchymal genes to determine the transcriptional profile of three different differentiation stages of EOC cells: primary EOC cells, MSFCs and sEOC cells (Fig. 1a, Supplementary Fig. 1, Supplementary Fig. 2a). We have observed that the mRNA expression of TWIST1 and CTNNB1 (mRNA of β-catenin) was upregulated in both MSFCs and sEOC cells (Fig. 1a, Sup. Table 1), indicating that the expression of TWIST1 and CTNNB1 was positively correlated and both were associated with mesenchymal differentiation. We have validated the array’s findings by detecting the mRNA expression levels of CTNNB1 and TWIST1 in the three stages of differentiation (Fig. 1b). Interestingly, CTNNB1 mRNA expression increased only in sEOC cells, while TWIST1 mRNA levels were increased in both MSFCs and sEOC cells (Fig. 1b). When we examined the protein expression levels, we observed high levels of β-catenin in primary EOC cells, and its expression was reduced in MSFCs and sEOC cells, which was negatively correlated with the protein level of TWIST1 (Fig. 1c).

**Figure 2:**
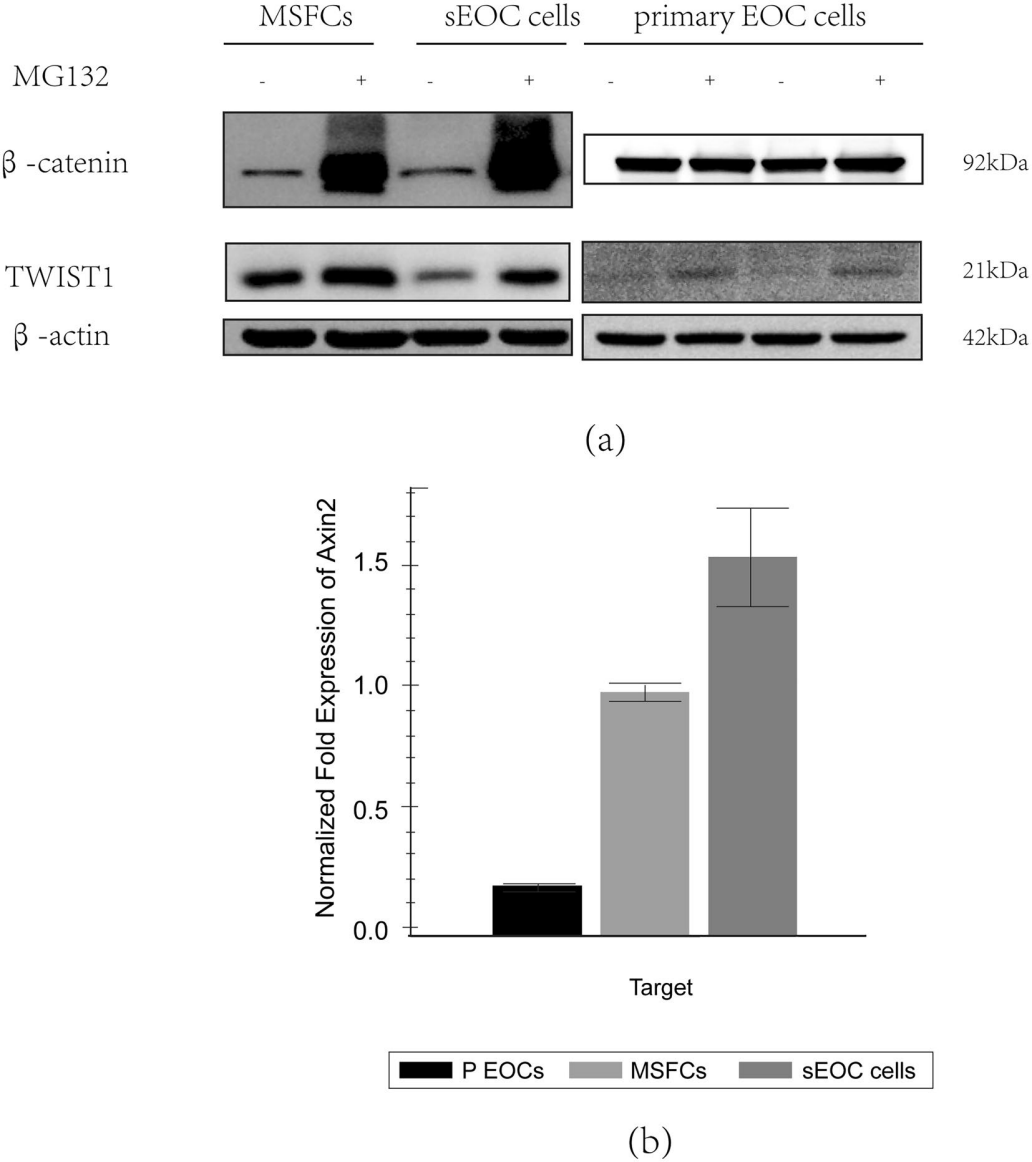
β-catenin is regulated by the protein degradation system in MSFCs and sEOC cells but not in primary EOC cells. (**a**)The levels of β-catenin protein in primary EOC cells, MSFCs and sEOC cells after treatment with MG132 (a protease inhibitor). (**b**)qPCR analysis of Axin2 mRNA in primary EOC cells, MSFCs and sEOC cells. Data are representative of three independent experiments.

Because β-catenin is degraded through the proteasome pathway^59^, we tested whether the decrease in β-catenin expression levels in the MSFCs and sEOC cells is due to proteosome degradation by treating the cells with MG132, a specific proteasome inhibitor. Interestingly, we found that the presence of MG132 restored the protein expression levels of β-catenin in MSFCs and sEOC cells, which were similar to those observed in EOCs (Fig. 2a), suggesting that β-catenin is degraded through the proteasome pathway during the differentiation into mesenchymal cells. These results also suggested that in primary EOC cells, there was an active mechanism preventing β-catenin degradation. Similar to previous reports, the presence of the proteasome inhibitor MG132 blocked TWIST1 degradation in all ovarian cancer cells, primarily in primary EOC cells that did not express TWIST1 (Fig. 2a).

To further validate the observation that β-catenin is degraded through the proteasome pathway during differentiation, we evaluated the expression levels of AXIN2, a component of the cytoplasmic destruction complex that controls β-catenin stability^19^. Our data showed differential Axin2 mRNA expression levels among primary EOC cells, MSFCs and sEOC cells (Fig. 2b), with the highest expression levels detected by qPCR in sEOC cells, which also showed the lowest expression of β-catenin (protein level) (Fig. 1c).Axin2 was weakly and negatively correlated with stem cell marker expression (Sup. Fig. 2b,c). Similarly, primary EOCs, which have the highest expression levels of β-catenin (Fig. 1c), showed low levels of AXIN2 (Fig. 2b). These results indicated that Axin2 was upregulated during the differentiation process.

### TWIST1 negatively regulated β-catenin expression in epithelial cells

**Figure 3:**
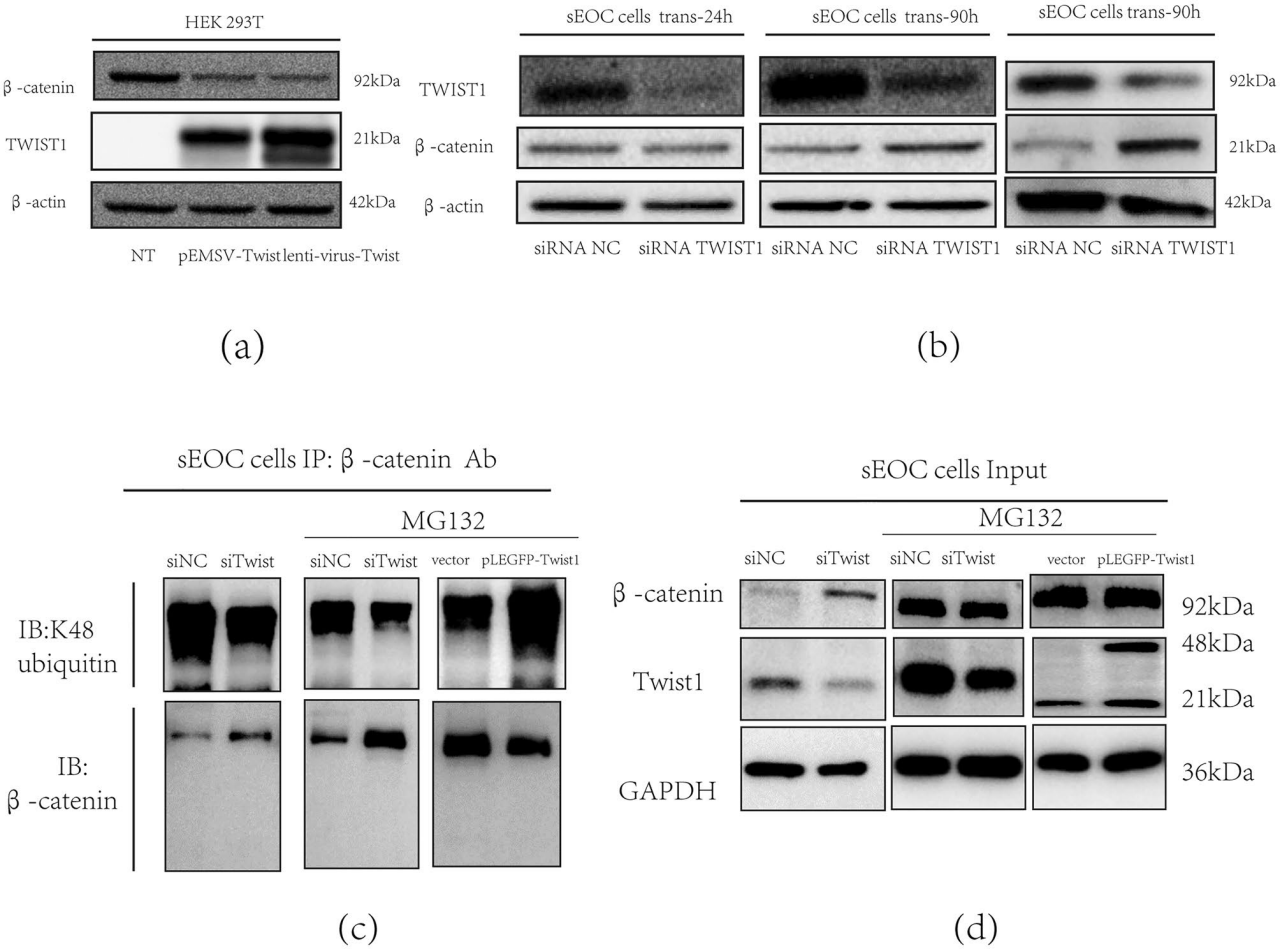
TWIST1 negatively regulates β-catenin by inducing its degradation. (**a**)β-catenin expression after overexpression of TWIST1 in normal epithelial cells. (**b**)β-catenin expression after TWIST1 knockdown in sEOC cells. (**c**)After transfection with vector, pEGFP-Twist1 or siTwist1, β-catenin degradation was examined by Western blotting of sEOCs. (**d**)After transfection with vector, pEGFP-Twist1 or siTwist1, β-catenin and TWIST1 were examined by Western blotting and sEOCs. Data are representative of three independent experiments.

Our next objective was to determine the factor(s) that regulate the expression of these components. We hypothesized that TWIST1 could be associated with the regulation of β-catenin expression. Consequently, we tested this hypothesis by using a coexpression system in HEK-293 T cells. Thus, we transfected HEK-293 T cells with a TWIST1-overexpressing plasmid (pEMSV-TWIST) in the presence of an β-catenin-expressing plasmid or empty vector control (p-lentivirus) and determined protein expression by western blotting. As shown in Fig. 3a, we observed that β-catenin expression when HEK293T cells were cotransfected with lentivirus control; however, β-catenin expression levels decreased in the presence of exogenous TWIST1 (Fig. 3a). To further determine the correlation between TWIST1 and β-catenin expression, we treated ovarian cancer cells with TWIST1 siRNA and measured the protein levels of β-catenin and TWIST1. Our data showed that inhibition of TWIST1 expression was associated with a time-dependent increase in β-catenin protein expression, further supporting the hypothesis that TWIST1 could significantly downregulate β-catenin (Fig. 3b). Therefore, we hypothesized that TWIST1 could induce the degradation of β-catenin. Therefore, the IP assay in Fig. 3c showed that TWIST1 positively regulated β-catenin ubiquitination degradation in sEOC cells. The IB assay of the input is shown in Fig. 3d.

### Twist1 negatively regulates β-catenin expression in sEOC cells by upregulating Axin2

**Figure 4:**
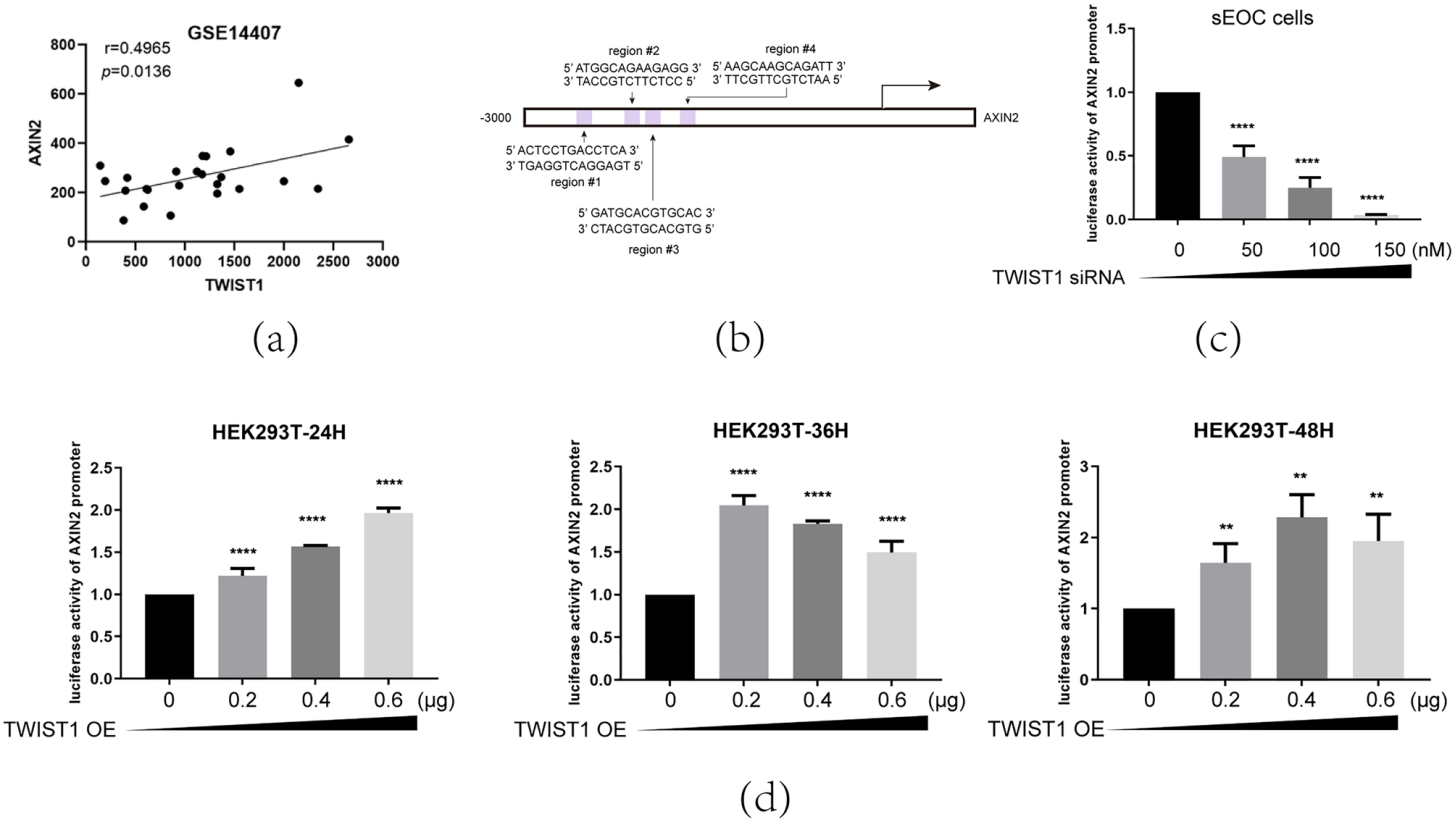
TWIST1 directly binds to Axin2. (**a**)Spearman correlation analysis of mRNA level of Twist1 and Axin2 levels in OC stem cells(n = 24). (**b**)The schematic structures of Twist1 putative binding sites in the AXIN2 promoter in EPD (https://epd.epfl.ch/). (**c**)Promoter luciferase reporter assays of Axin2 in sEOC cells with TWIST1 knockdown (siTwist1, + 50 nM, +  + 100 nM, +  +  +  + 150 nM). **** *p*,** *p* < 0.05, Student’s t test. (**d**)Promoter luciferase reporter assays of Axin2 in normal epithelial cells with TWIST1 overexpressed, oeTWIST1 by transfecting PCMV-SPORT6-Twist1 (oe TWIST1: 0.2 μg/per 24 well, 0.4 μg/per 24 well, 0.6 μg/per 24 well). ****p,** *p* < 0.05, Student’s t test.

To further study the mechanism by which TWIST1 may regulate β-catenin, we tested whether AXIN2, a component of the degradation complex of β-catenin^58^, is regulated by TWIST1. Since the mRNA levels of TWIST1 and AXIN2 are positively correlated in samples from the GSE14407 dataset (Fig. 4a) and the AXIN2 promoter contains four putative TWIST1 binding regions in https://epd.epfl.ch/ (Fig. 4b). However, TWIST1 is no significant correlation with stem cell markers (Sup. Fig. 2b,c). We evaluated whether TWIST1 could directly bind to the AXIN2 promoter by using a dual-luciferase reporter system. Thus, the AXIN2 promoter was cloned into the pGL3-basic vector and transfected into sEOC cells, which constitutively express high levels of the TWIST protein, or into sEOC cells-TWIST KD (transfected with siTWIST1). Compared to sEOC cells (high TWIST expression), the luciferase activity driven by the AXIN2 promoters was considerably decreased by inhibition of TWIST1 expression (Fig. 4c). On the other hand, overexpression of TWIST1 in 293 T cells enhanced luciferase activity when cotransfected with the AXIN2 promoter (Fig. 4d).

**Figure 5:**
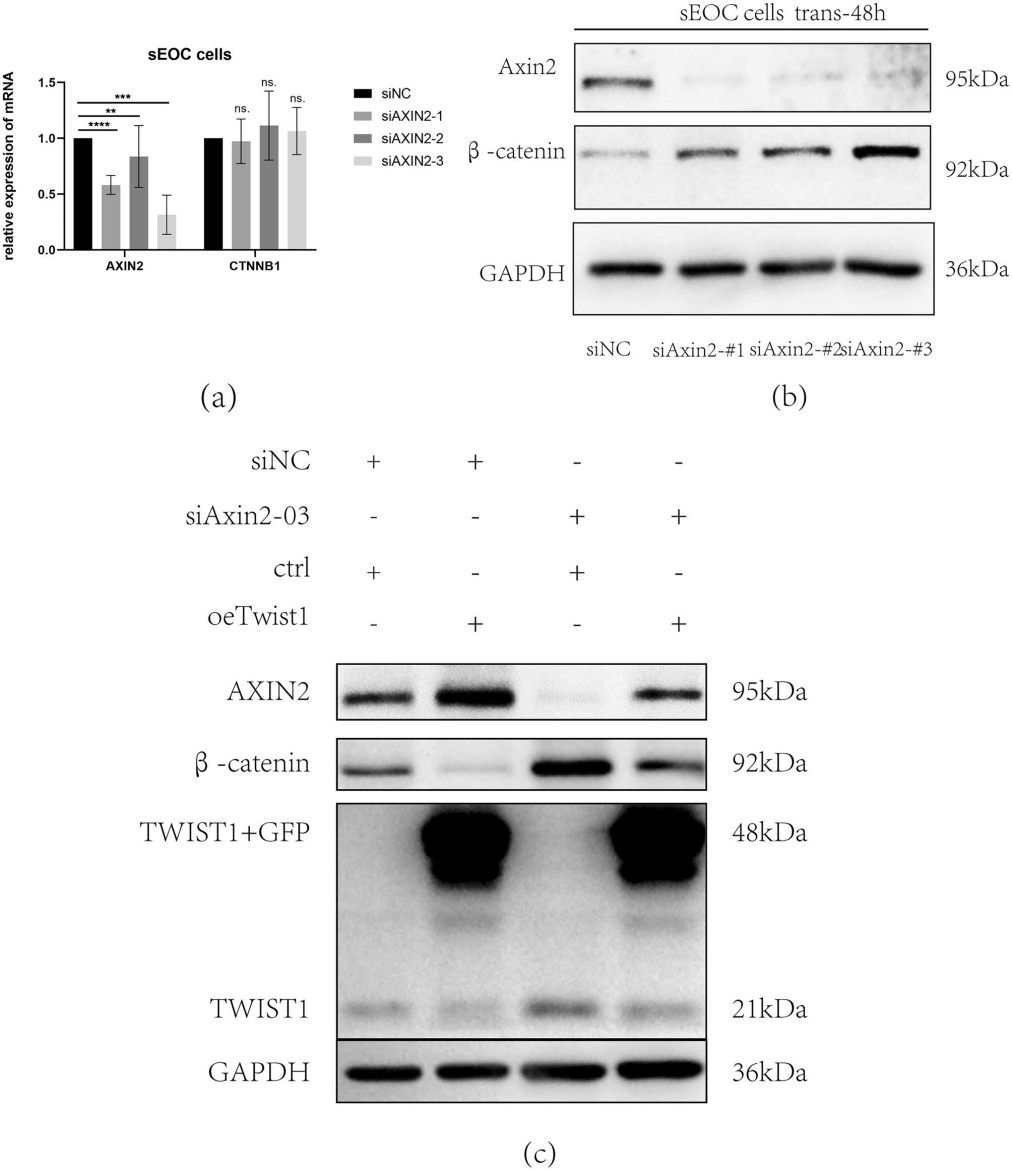
AXIN2 promotes the degradation of β-catenin. (**a**) Efficiency of AXIN2 knockdown and mRNA level of CTNNB1 in sEOC cells. **** *p*,*** *p*,** *p* < 0.05, Student’s t test. (**b**) Expression of AXIN2 and β-catenin at the protein level after AXIN2 knockdown in sEOC cells. (**c**) Expression of AXIN2, β-catenin and TWIST1 after transfection with siAXIN2 and TWIST1. Data are representative of three independent experiments.

Having demonstrated that TWIST1 can regulate AXIN2 expression, we next determined whether AXIN2 is the mediator of TWIST1-induced β-catenin degradation. We knocked down AXIN2 by siRNA and then detected the mRNA levels of Axin2 and CTNNB1 (Fig. 5a). We found that si Axin2-01, -02 and -03 significantly knocked down Axin2 levels without affecting CTNNB1 expression. However, we found that β-catenin was upregulated at the protein level upon AXIN2 knockdown (Fig. 5b). Furthermore, we performed rescue experiments (Fig. 5c) and found that after knockdown of AXIN2, the reduction in β-catenin induced by TWIST1 was diminished, which proved that AXIN2 is an essential factor in the TWIST1/AXIN2/β-catenin axis. These results indicated that knockdown of AXIN2 exerts a regulatory role not on the transcription of CTNNB1 but on the prevention of degradation at the protein level.

## Discussion

Previously, we reported that different ovarian cancer cell types are derived from CD44^+^/MyD88^+^ EOC stem cells with lost stemness^17,60^. Primary EOC cells could be a source of ovarian cancer metastasis by generating MSFCs with enhanced migratory capacity and ability to recreate an epithelial ovarian cancer tumor in a secondary site, which is the result of EMT and is regulated by TWIST1^26^. However, our in vitro cell models were from ascites obtained from patients diagnosed with stage III/IV serous ovarian carcinoma, which only contained one particular ovarian cancer type and did not represent all histotypes of ovarian cancer, which is also a limitation of our study. It would be better if we are able to derive cells from different patients.The transition of EMT and CSCs is a dynamic process, and epithelial ovarian cancer stem-like cells also undergo EMT, a transdifferentiation process. The final step of the establishment of tumors at secondary metastatic sites involves MET (mesenchymal-epithelial transition), which initiates “secondary” epithelial ovarian cancer cells (sEOC cells)^20^. In the differentiation process of primary EOC cells to sEOC cells, genes expression were significantly changed.

TWIST1 promotes cancer metastasis by regulating epithelial mesenchymal transition (EMT) and is critical for the maintenance of EMT-associated CSC-like characteristics^61,62^. Transient overexpression of TWIST1 activates a subset of mammary epithelial cells with stem cell-like properties and the whole process of cancer metastasis^31^. A subsequent study reported that TWIST1 induced EMT and a cancer stem-like cell phenotype, contributing to irinotecan resistance and promoting the migration of colon cancer and invasion^61^. The stability of TWIST1 is a vital step in maintaining the cancer stem-like cell features of castration-resistant prostate cancer^63^.

However, in-depth research on TWIST1 and cancer stem cell differentiation is poorly reported. To investigate the role of TWIST1 during the differentiation process of ovarian cancer stem-like cells, we performed an EMT array in primary EOC cells, MSFCs, and sEOC cells. We found that the expression of TWIST1 increased as the ovarian cancer cells differentiated, which supported the conclusion we reported before^26^. Meanwhile, the mRNA expression of CTNNB1 is positively correlated with TWIST1. However, CTNNB1 is positively associated with cancer stemness^64^. Therefore, we detected the protein level of β-catenin. Interestingly, the protein level of β-catenin is inversely associated with its mRNA level in 3 cell lines and is the highest in primary EOC cells, which is consistent with other research^64^.

Because the mRNA level of CTNNB1 is high while the protein level is low in sEOC cells and MSFCs, we considered that β-catenin may be degraded during these stages. We next used MG132 to block the proteasome degradation system of β-catenin in 3 cell lines. After treatment with MG132, β-catenin was upregulated in MSFCs and sEOC cells, which supported the traditional way of β-catenin degradation^59^. Interestingly, in primary EOC cells, β-catenin is not regulated by the above protein degradation system, indicating that β-catenin was not degraded before. We hypothesized that TWIST1 may regulate the degradation of β-catenin because in primary EOC cells, TWIST1 was degraded and lost the function of promoting the degradation of β-catenin, thus preventing β-catenin from increasing even after treatment with the protease inhibitor MG132. We further proved that TWIST1 induced ubiquitination of β-catenin, a classical β-catenin degradation pathway, in sEOC cells. The destruction complex of β-catenin contains AXIN2, which participates in a negative feedback loop that reduces the duration or severity of the Wnt start signal^58,65–68^. In our study, we downregulated Axin2 and detected the mRNA and protein levels of β-catenin and found that knockdown of AXIN2 did not affect the transcription of CTNNB1 but drastically increased the expression of β-catenin, which indicated that AXIN2 promoted the degradation of β-catenin and supported the findings of other studies.

We next detected the mRNA level of AXIN2 in 3 cell lines and observed that in primary EOC cells, in which β-catenin is not degraded by the proteasome pathway, the mRNA level of AXIN2 is the lowest compared with that in MSFCs and sEOC cells. The subsequent question is: What increases AXIN2 expression? Can TWIST1 regulate its expression? Way T D reported that in head and neck squamous cell carcinoma (HNSCC), overexpression of TWIST1 upregulates β-catenin expression^69^. Ming Tan identified that the FZD7-TWIST1 axis is critical for ovarian carcinoma tumorigenesis and anoikis resistance^70^. Chang YW reported that downregulated TWIST1 upregulated β-catenin with induction of phenotypic elevation of CSCs. In the EMT process, they found that TWIST1 interacted with β-catenin to enhance the transcriptional activity of the β-catenin/TCF4 complex, including binding to the CSC marker ABCG2^71^. Collectively, previous studies have not clearly demonstrated the mechanism between TWIST1 and β-catenin. To explore whether TWIST1 could regulate β-catenin, we uncovered the negative role of TWIST1 in the expression of β-catenin at the protein level during the process of ovarian cancer stem cell differentiation. We showed that TWIST1 negatively regulated β-catenin expression in normal epithelial cells, while knockdown of TWIST1 upregulated β-catenin in sEOC cells. We hypothesized that TWIST1 may regulate Axin2 expression. Then, we used a dual-luciferase assay to confirm the direct binding of TWIST1 to the promoter region of AXIN2.

**Figure 6:**
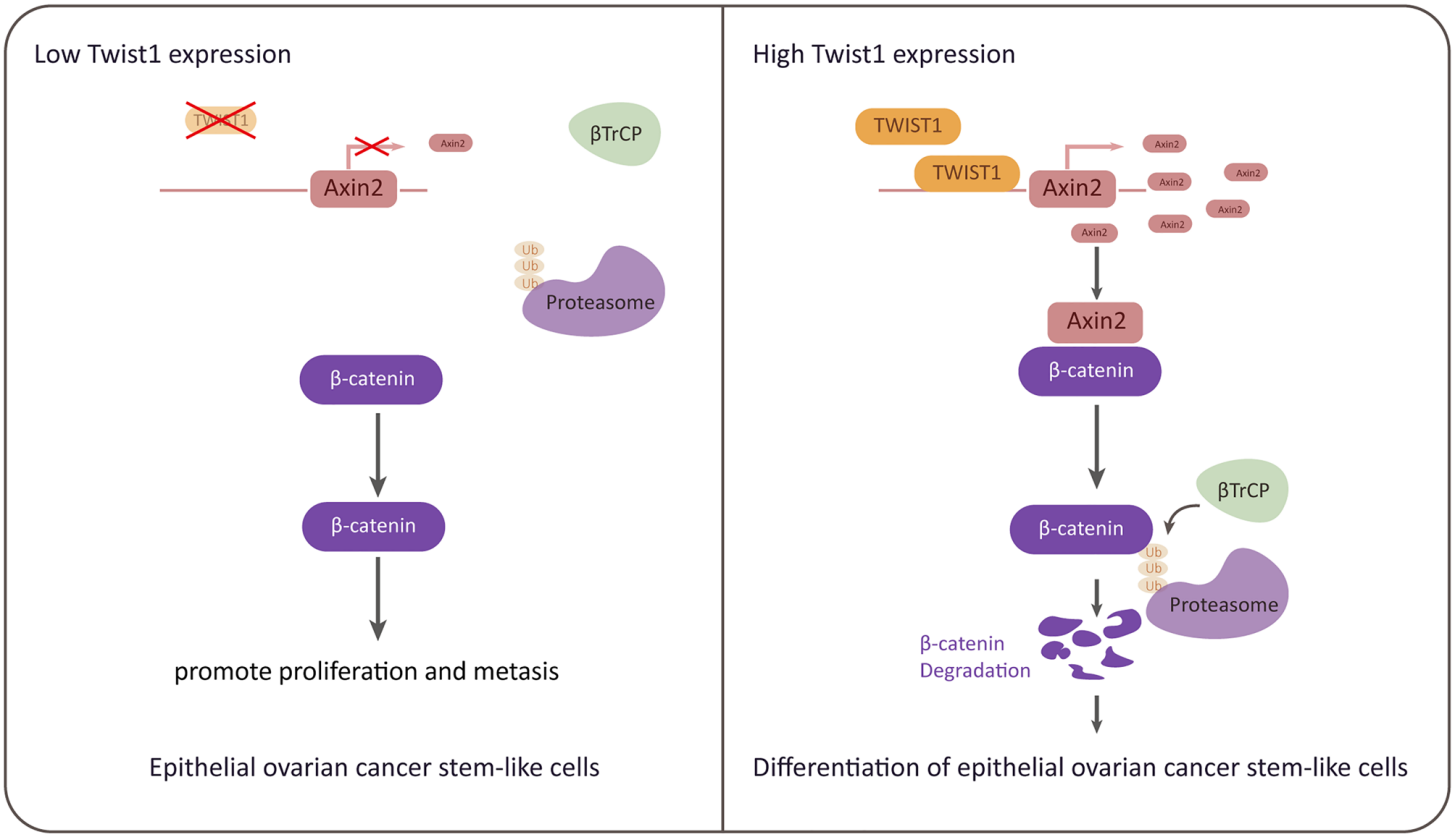
Working model of TWIST1 functions.

In conclusion, we demonstrate that the degradation of β-catenin is differentially regulated in ovarian cancer stem-like cells compared to non-stem ovarian cancer cells. β-catenin downregulation and degradation are observed in non-stem ovarian cancer cells and are positively correlated with TWIST1, which promotes the upregulation of AXIN2, one of the components of the β-catenin destruction complex. Moreover, we detected that TWIST1 could negatively regulate β-catenin by directly binding to AXIN 2. This is the first study to unveil the relationship between TWIST1 and AXIN2 and provides a new perspective to understand the differentiation process of ovarian cancer. (Fig. 6).

## Materials and methods

### Reagents and antibodies

MG132 (#C2211) was purchased from Sigma (St. Louis, MO, USA). Beta-actin antibody was purchased from Sungene Biotech (Tianjin, China) clone KM9001. Beta-catenin and GAPDH antibodies were purchased from Proteintech and Utibody, respectively (10366-1-AP and UM4002, respectively). TWIST antibody was purchased from Santa Cruz (sc-81417). Ubiquitin K48 antibody was purchased from ZENBIO (R24785).

### Cell lines and cell culture

HEK-293 T cell lines were purchased from American Type Culture Collection (ATCC; http://www.atcc.org/). EOC cell lines were used: primary epithelial ovarian cancer cells (primary EOC cells), mesenchymal spheroid-forming cells (MSFCs) and secondary epithelial ovarian cancer cells (sEOC cells).

Primary EOC cells were isolated from ascites obtained from patients diagnosed with stage III/IV serous ovarian carcinoma, and their characterization has been previously reported by our group^15,16,18,19,21,22,72,73^. STR profiling was performed every year, and mycoplasma testing was performed every month. In this study, these cell lines are designated “primary” EOC cells. All patients signed consent forms, and the use of patient samples was approved by Yale University’s Human Investigations Committee (HIC no. 10425). Cells were grown in RPMI media supplemented with 10% FBS, 1000 U/ml penicillin, 100 µg/ml streptomycin, 10 mM HEPES, 100 nM nonessential amino acids, and 1 mM sodium pyruvate and cultured at 37 °C with 5% CO_2_. 3D spheroids derived from primary EOC cells as previously described^19,20,22,24,26,60,74^. Briefly, cells were maintained in high confluence in low-serum conditions (1% fetal bovine serum) until multiple foci underwent morphological changes to a fibroblastic phenotype, typically after 2 weeks in culture. Media collection followed by centrifugation isolated cells that detached. Cell pellets were resuspended in growth media and cultured in ultralow attachment plates (Corning Life Sciences, Corning, NY) for 5 days to promote spheroid formation. The resulting spheroids (MSFCs) are typically not uniform in size but exhibit a compacted morphology with a distinct outer layer. Without any further selection, 5-day-old spheroids were transferred to tissue culture-treated flasks, allowed to reattach, and passaged five times. The resulting sEOC cells at p5–p10 were used for the experiments.

### Plasmid and siRNA transfection

Transfection of plasmid and siRNA was performed as previously described^20^. pEMSV-TWIST1 was provided by Dr. Ernst-Martin Fuchtbauer, University of Aarhus, Denmark, and pFUGW was provided by Dr. Wange Lu, University of Southern California, USA. The human gene promoter region generated by PCR amplification from HEK-293 T cells was cloned into the pGL3-basic luciferase reporter plasmid (Promega, Madison, WI, USA). Primer-F: ggggtaccgaacccgggattgttgtttcccgc, primer-R: ctagctagccagaagatcctggcccaagagaagc. The cDNA encoding TWIST1 was obtained by PCR amplification from sEOC cells and cloned into pEGFP-c1. Primer-F: cggaattctatgatgcaggacgtgtcca, primer-R: CGCGGATCCtagttatccagctccagagtctcta. TWIST1-siRNA and AXIN2-siRNA were purchased from RiboBio, Guangzhou, China.

### Western blotting and RT-qPCR

Western blotting and RT-qPCR were performed as previously described^20^.

### IP assay

sEOC cells were seeded in 10 cm cell culture dishes, transfected with oeTWIST1 plasmid and siTWIST1 siRNA with or without MG132, and lysed with 1 ml IP lysis buffer. The cell lysis was rotated and mixed 12 h with beta-catenin antibody 4 °C before prewashed Protein A/G Magnetic beads (Santa Cruz Biotechnology) were added and then incubated upside down for at least 1 h at 4 °C. Finally, the interaction protein was pulled down by the magnetic beads. The magnetic beads were resuspended in SDS-loading buffer and then heated up to 96 °C for 10 min before loaded to gel. For the ubiquitination assays, An K48-ubiquitin antibody was used to detect the ubiquitination.

### Luciferase reporter assay

Luciferase reporter assay as previously described^75^.

### EMT array

Total RNA was prepared from three EOC cell lines, primary EOC cells (primary EOC cells), mesenchymal spheroid-forming cells (MSFCs) and secondary epithelial ovarian cancer cells (sEOC cells), using the RNeasy Mini kit (Qiagen, Valencia, CA, USA). Total RNA isolated from each sample was then used as a template for cDNA synthesis and prepared with an RT^2^ first strand kit (SABioscience, no. C-03). Total cDNA was used as a template for the EMT array using an RT^2^ Profiler™ PCR Array Human Epithelial to Mesenchymal Transition (EMT) plate (SABioscience, no. PAHS-090A). This plate was precoated with 42 pairs of primers for 42 kinds of genes (included in Supplementary Table 1). The expression levels of various genes were assessed by real-time PCR amplification with PCR Master Mix RT^2^ Real-Time SYBR Green/ROX (SABioscience, no. PA-012) using the ABI 7500 Real-Time Standard Cycler (Applied Biosystems, Foster City, CA, USA). Validation of the gene array was performed in five cell cultures for each subtype of cells (n = 15)*.*

### Database analyses

A cohort of ovarian cancer data was downloaded from the GEO database (http://www.ncbi.nlm.nih.gov/geo/): GSE14407^76^. The putative binding site of AXIN2 and TWIST1 is https://epd.epfl.ch/.

### Statistical analysis

Data are expressed as the mean ± standard error. Statistical significance (*p* < 0.05) was determined using either two-tailed unpaired t-tests or the Mann–Whitney U test for nonparametric data. Spearman correlation analysis of TWIST1/SOX2/ALDH1A3 and Axin2 levels by GraphPad Prism 9.0.

## Supplementary Information

Supplementary Information 1.

Supplementary Information 2.

Supplementary Information 3.

Supplementary Information 4.
