# Supplementary material for: TWIST1 induces proteasomal degradation of β-catenin during the differentiation of ovarian cancer stem-like cells
Source: Sci Rep. 2022 Sep 19;12:15650. doi: 10.1038/s41598-022-18662-2 (PMC9485151; doi:10.1038/s41598-022-18662-2)
Supplement: Supplementary file 1 — Supplementary Information 1. [file 41598_2022_18662_MOESM1_ESM.pdf]

Supplementary Fig.3
original images of western blots

Fig2a MSFCs and sEOC cells

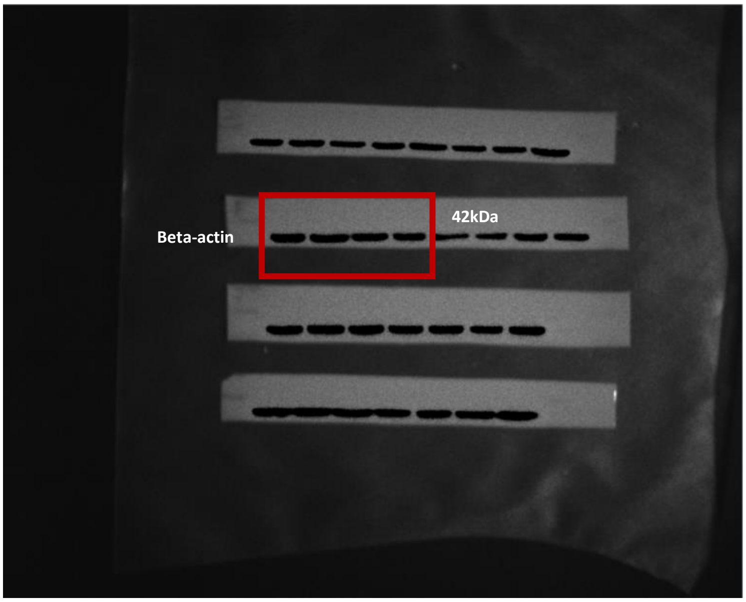

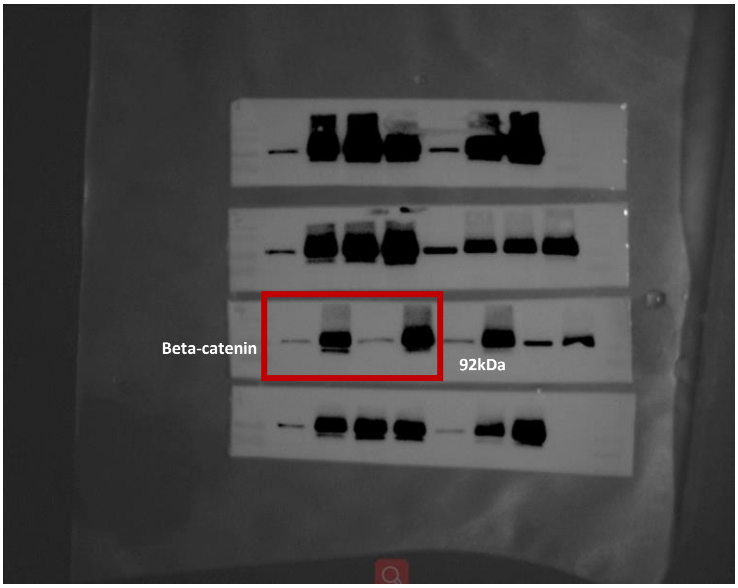

Fig2a MSFCs and sEOC cells

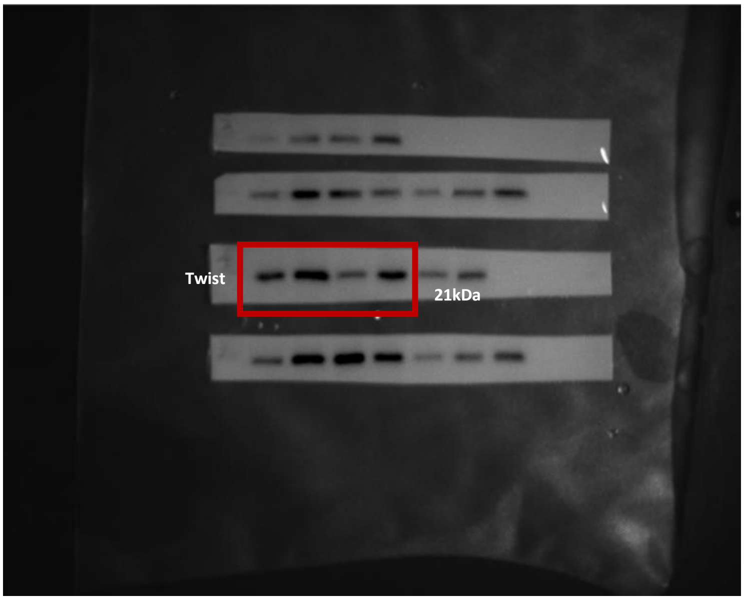

Fig3c. K48

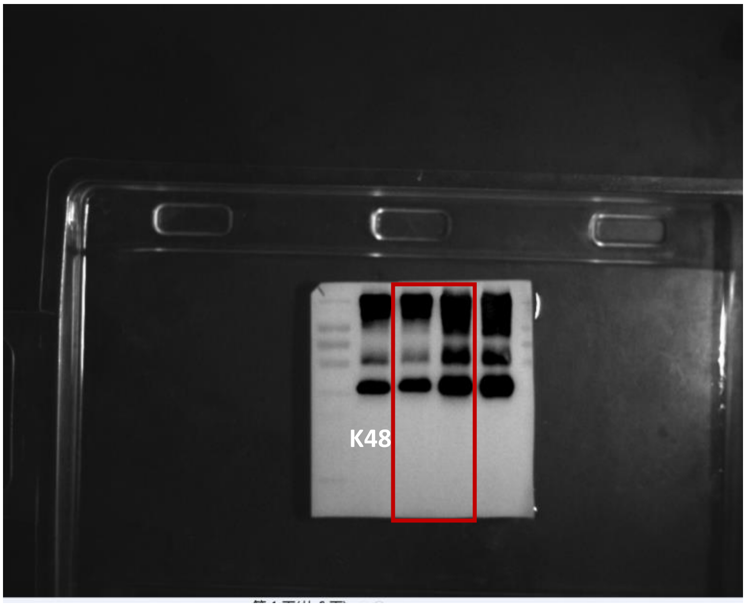

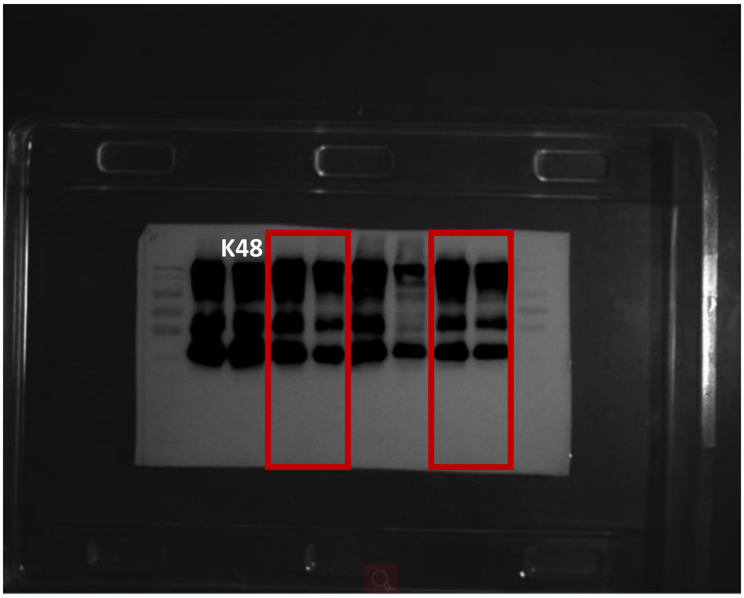

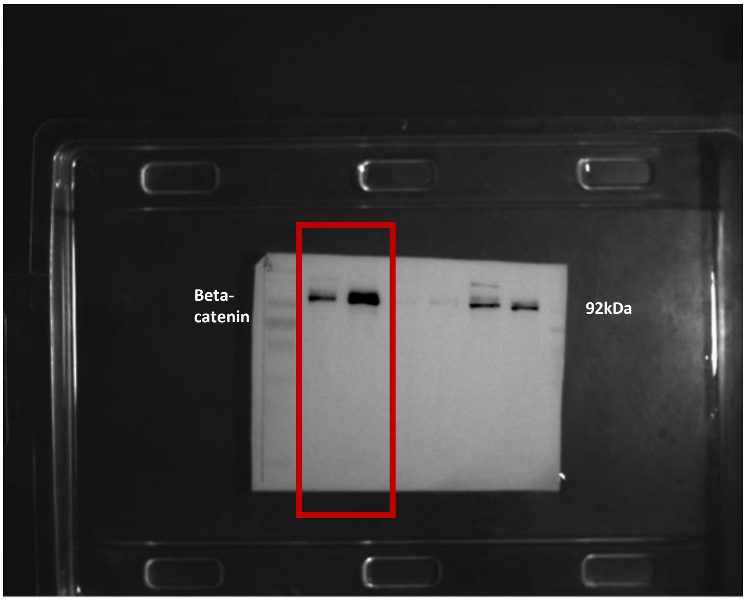

Fig3c.Beta-catenin

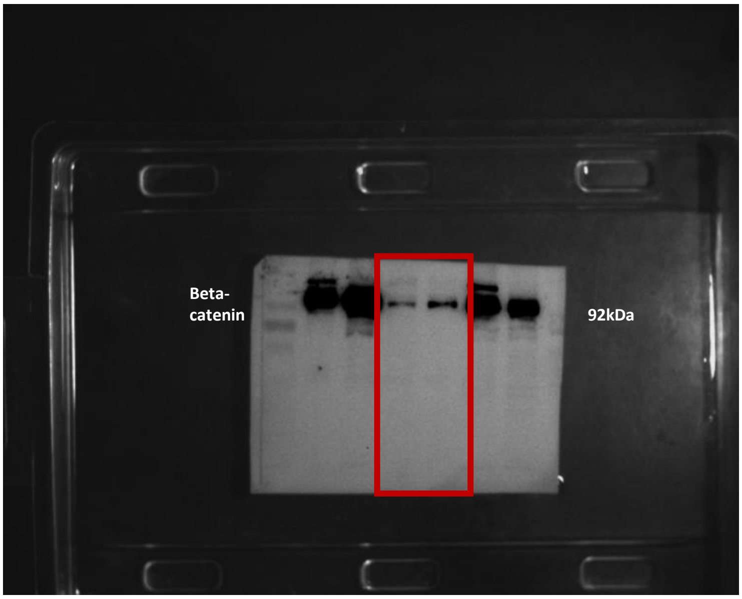

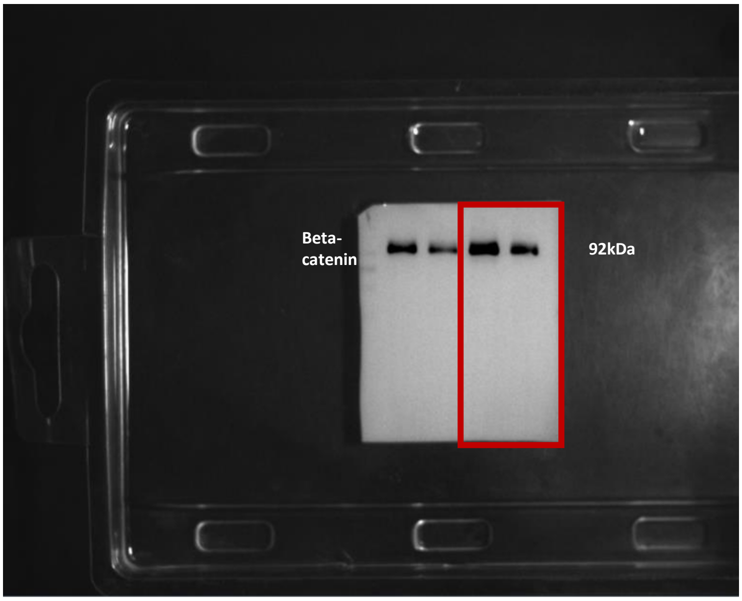

Fig3d.

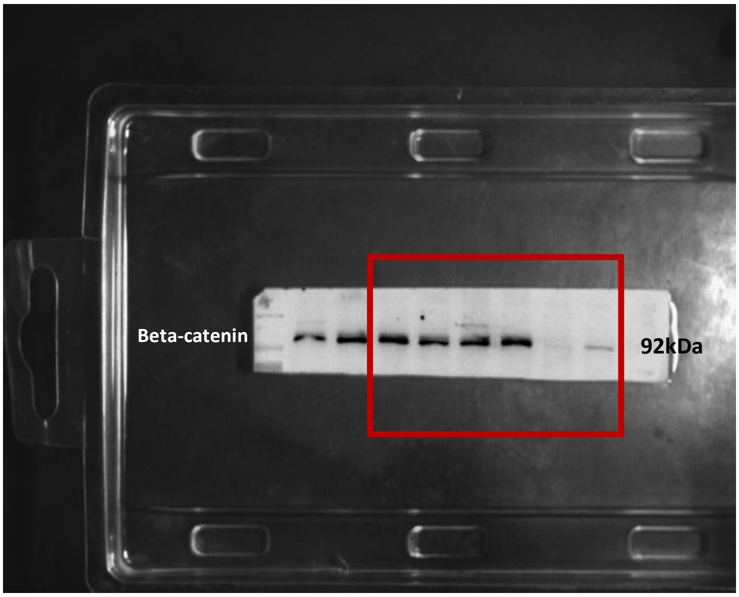

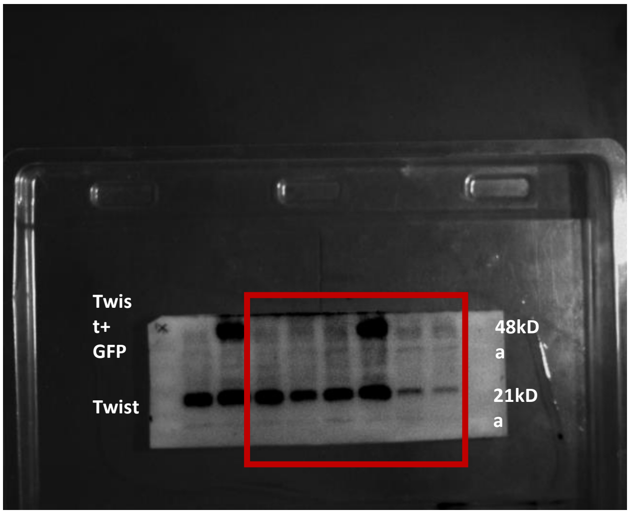

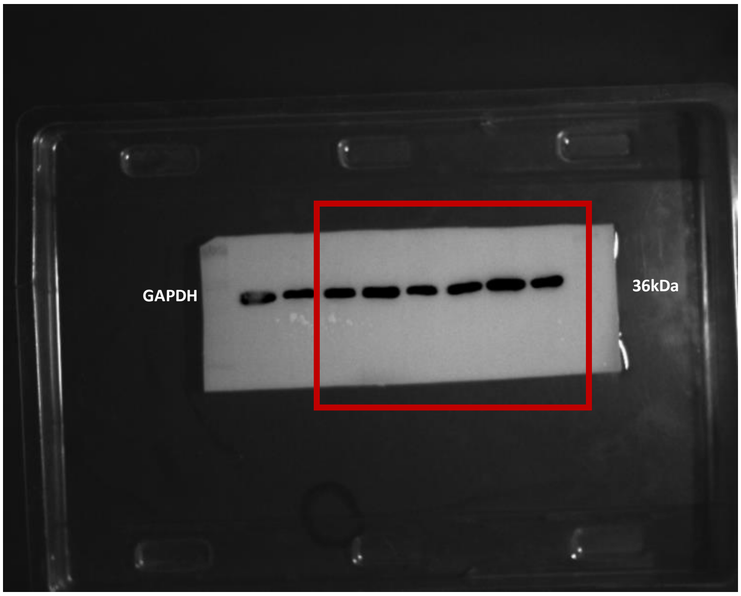

Fig5b

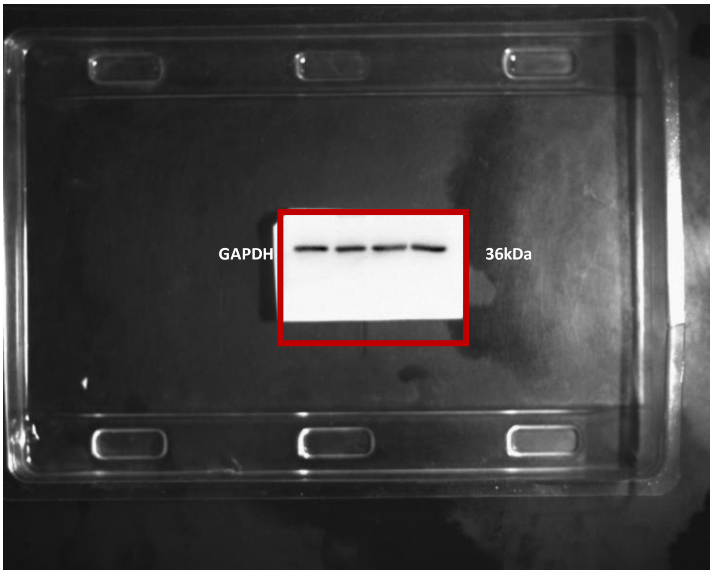

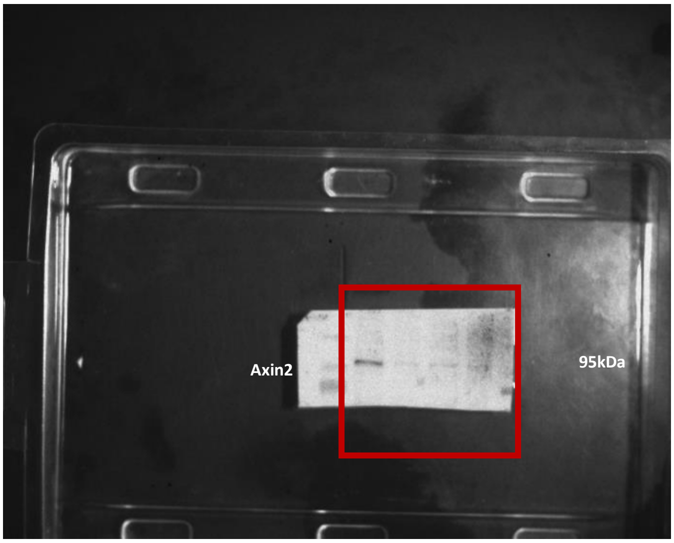

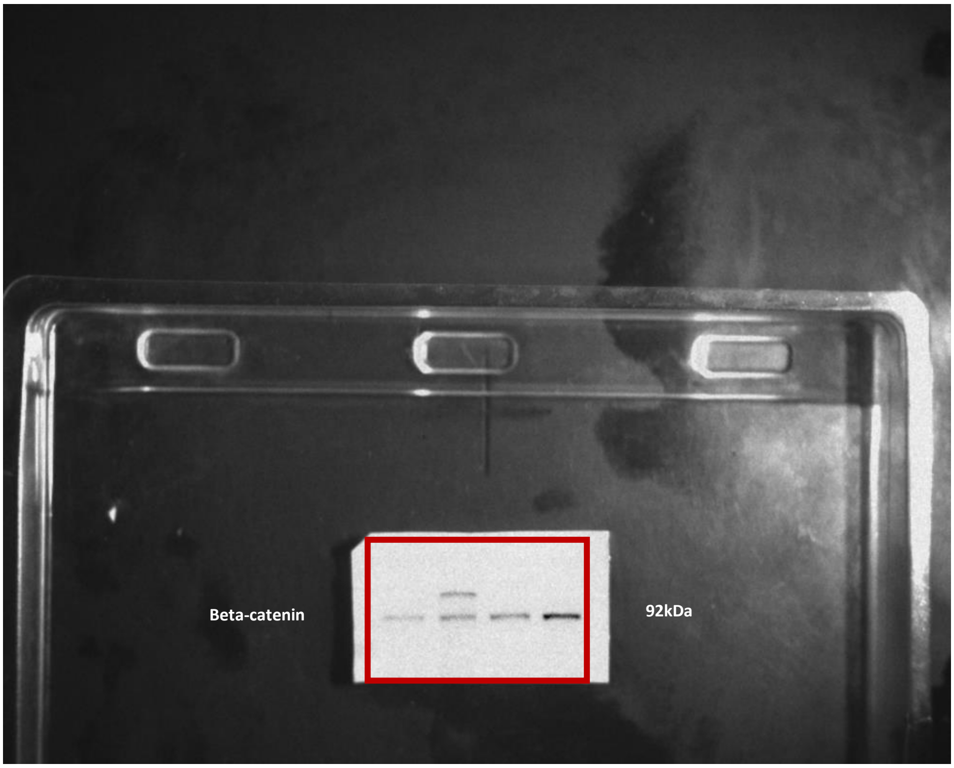

Fig5c

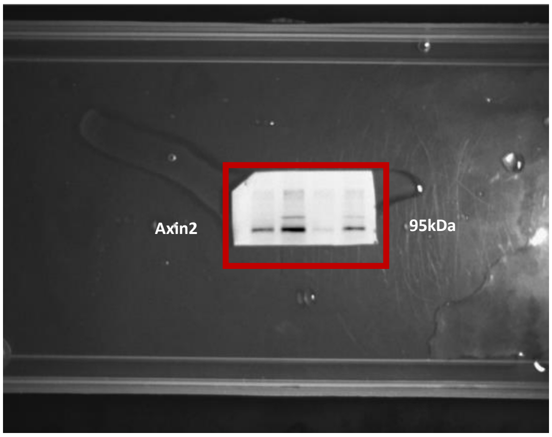

Axin2
95kDa

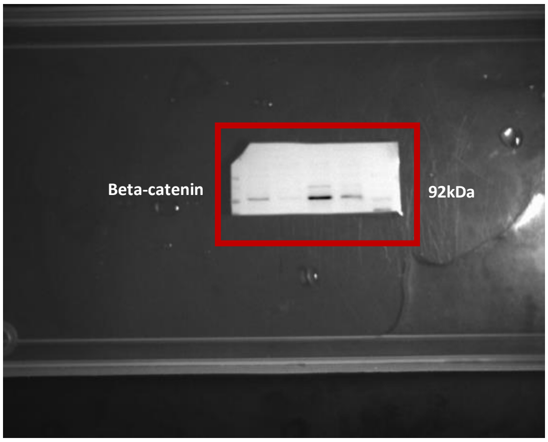

Beta-catenin
92kDa

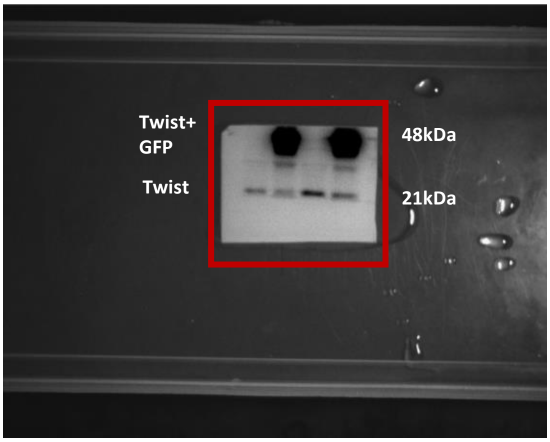

Twist+
GFP
Twist
48kDa
21kDa

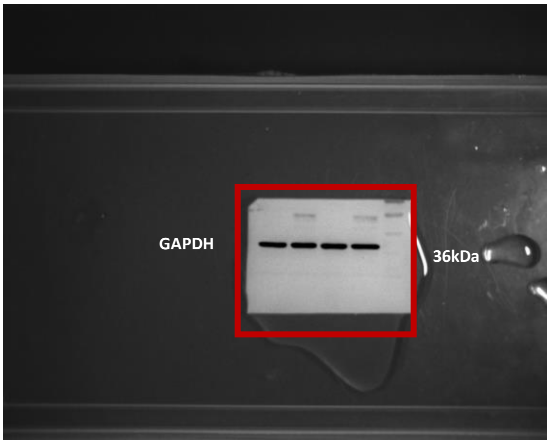

GAPDH
36kDa

- We added the original, unprocessed images of western blots(Fig2a,Fig3c,Fig3d,Fig5b,Fig5c) in supplementary material.
- Unfortunately, The western blots did in Yale University are missing because of the work mobility(GangYin came back to China from Yale University, Gil Mor moved to Wayne State University from Yale University).
