# Supplementary figures and images for: TWIST1 induces proteasomal degradation of β-catenin during the differentiation of ovarian cancer stem-like cells
Source: Sci Rep. 2022 Sep 19;12:15650. doi: 10.1038/s41598-022-18662-2 (PMC9485151; doi:10.1038/s41598-022-18662-2)

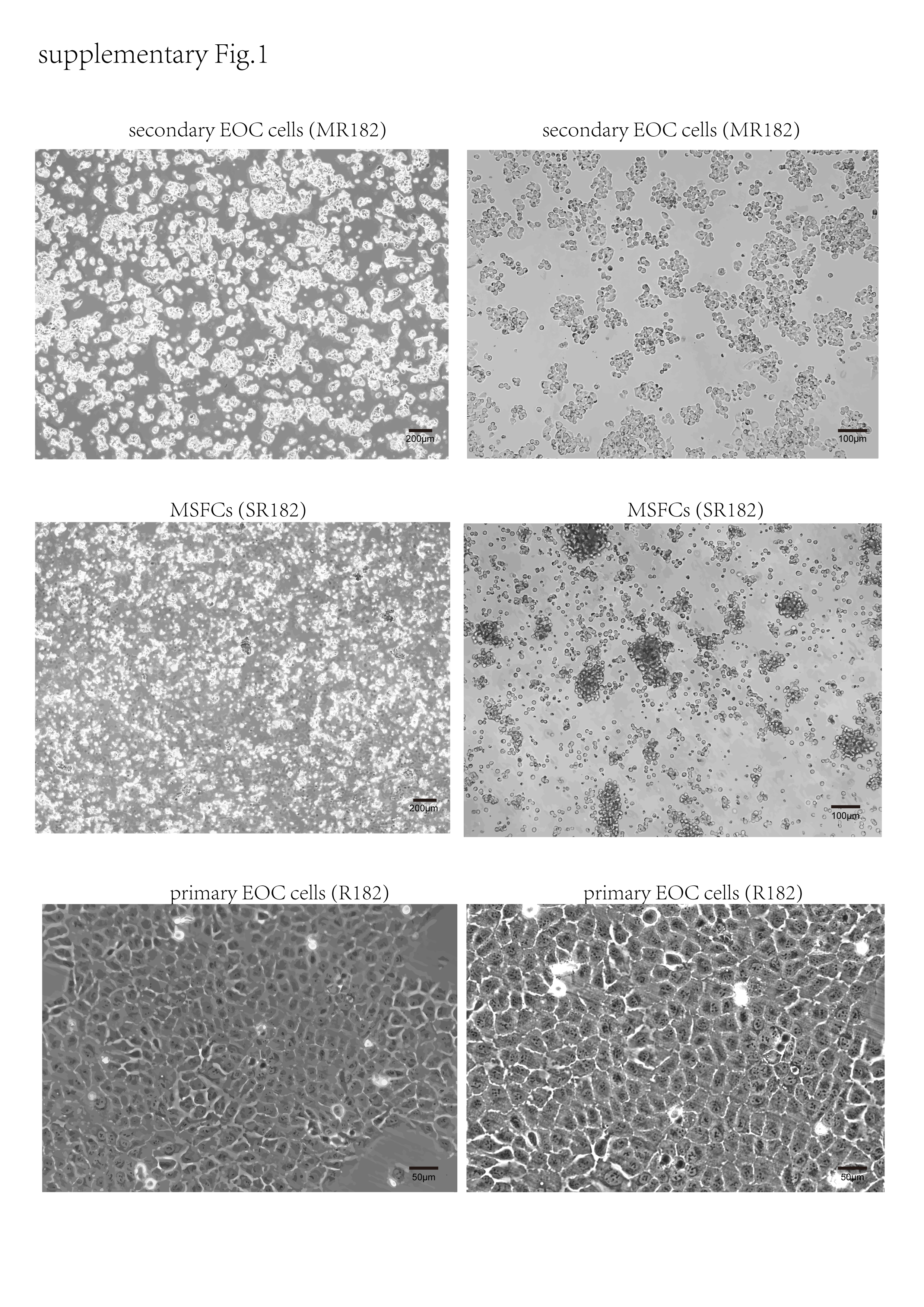

Supplement: Supplementary file 2 — Supplementary Information 2. [file 41598_2022_18662_MOESM2_ESM.tif]

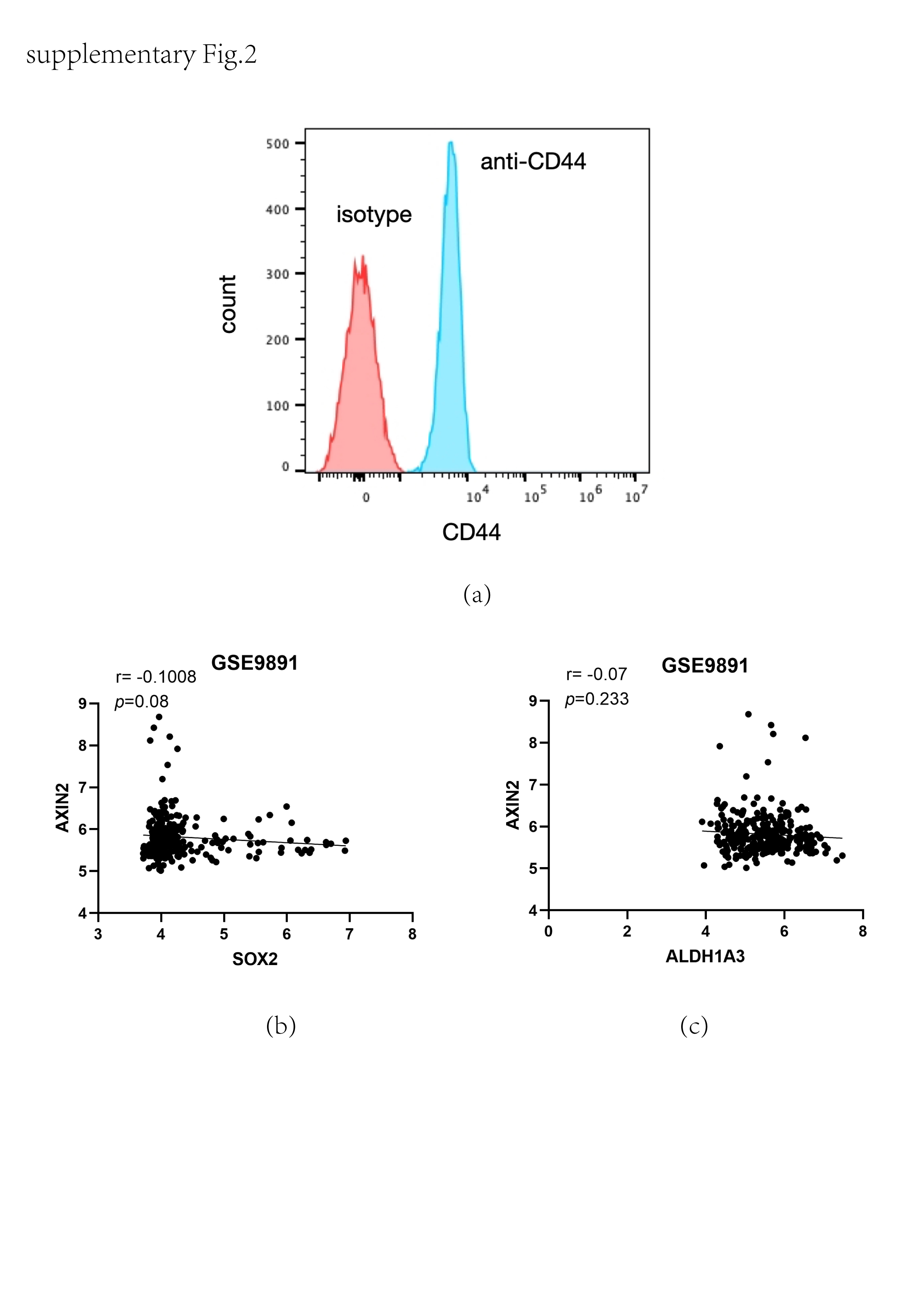

Supplement: Supplementary file 3 — Supplementary Information 3. [file 41598_2022_18662_MOESM3_ESM.tif]
