# Supplementary material for: TWIST1 induces proteasomal degradation of β-catenin during the differentiation of ovarian cancer stem-like cells
Source: Sci Rep. 2022 Sep 19;12:15650. doi: 10.1038/s41598-022-18662-2 (PMC9485151; doi:10.1038/s41598-022-18662-2)
Supplement: Supplementary file 4 — Supplementary Information 4. [file 41598_2022_18662_MOESM4_ESM.docx]

Supp. Table.1. Gene expression profile of EMT array

|  | Primary EOC cells (R182) | | MFSCs (SR182) | Secondary EOC cells (MR182) |
| --- | --- | --- | --- | --- |
| UP | KRT19,MITF,NUDT13,  F11R,ITGB1,TSPAN13,  MSN,FFBP1,DSP,VCAN,  EGFR,CAV2,KRT,MST1R,  TFP12,SIPI,GSK3B,  PPPDE2,MMP3,SPP1 | | MMP9,SOX10,FOXC2,  KRT14,TIMP1,TGFB3,  COL1A2,SNAI2,TGFB2, MMP3,SPP1,IL1RN,  MMP2 | COL5A2,TGFB1,TWIST1,  BMP7,TGFB2,SPARC,TCF4,  VIM,COL3A1,CTNNB1,GCS,SNAI3, SIPI,GSK3B, |
| DOWN | MMP9,SOX10,FOXC2,  KRT14,TIMP1,TGFB3,  COL1A2,SNAI2,COL5A2,  TGFB1,TWIST1,BMP7,  TGFB2,SPARC,TCF4,  VIM,COL3A1,CTNNB1,  GCS,SNAI3,MMP2 | | KRT19,MITF,NUDT13,  F11R,ITGB1,TSPAN13,  MSN,FGFBP1,DSP,  VCAN,EGFR,CAV2,KRT,  MST1R,TFP12,COL5A2,  TGFB1,TWIST1,SPARC,  TCF4,VIM,COL3A1,  CTNNB1,GCS,SNAI3,  SIPI,GSK3B,PPPDE2 | MMP9,SOX10,FOXC2,KRT14  TGFB3,COL1A2,SNAI2,  KRT19,MITF,NUDT13,F11R,  ITGB1,TSPAN13,MSN,FFBP1DSP,VCAN,EGFR,CAV2,KRT,  MST1R,TFP12, MMP3,SPP1,  IL1RN, MMP2 |
| AVERAGE | IL1RN | BMP7, | | TIMP1, PPPDE2 |

Red shows up-regulation; Green shows down-regulation.
